# Hepatitis B and C Co-Infection in HIV Patients from the TREAT Asia HIV Observational Database: Analysis of Risk Factors and Survival

**DOI:** 10.1371/journal.pone.0150512

**Published:** 2016-03-02

**Authors:** Marcelo Chen, Wing-Wai Wong, Matthew G. Law, Sasisopin Kiertiburanakul, Evy Yunihastuti, Tuti Parwati Merati, Poh Lian Lim, Romanee Chaiwarith, Praphan Phanuphak, Man Po Lee, Nagalingeswaran Kumarasamy, Vonthanak Saphonn, Rossana Ditangco, Benedict L. H. Sim, Kinh Van Nguyen, Sanjay Pujari, Adeeba Kamarulzaman, Fujie Zhang, Thuy Thanh Pham, Jun Yong Choi, Shinichi Oka, Pacharee Kantipong, Mahiran Mustafa, Winai Ratanasuwan, Nicolas Durier, Yi-Ming Arthur Chen

**Affiliations:** 1 Center for Infectious Disease and Cancer Research, Kaohsiung Medical University, Kaohsiung, Taiwan; 2 Department of Urology, MacKay Memorial Hospital, Taipei, Taiwan; 3 Department of Cosmetic Applications and Management, MacKay Junior College of Medicine, Nursing and Management, Taipei City, Taiwan; 4 Department of Infectious Diseases, Taipei Veterans General Hospital, Taipei, Taiwan; 5 The Kirby Institute, UNSW Australia, Sydney, Australia; 6 Division of Infectious Diseases, Department of Medicine, Ramathibodi Hospital, Mahidol University, Bangkok, Thailand; 7 Working Group on AIDS Faculty of Medicine, University of Indonesia/Cipto Mangunkusumo Hospital, Jakarta, Indonesia; 8 Faculty of Medicine Udayana University & Sanglah Hospital, Bali, Indonesia; 9 Tan Tock Seng Hospital, Singapore, Singapore; 10 Research Institute for Health Sciences, Chiang Mai, Thailand; 11 HIV-NAT/ Thai Red Cross AIDS Research Centre, Bangkok, Thailand; 12 Queen Elizabeth Hospital, Hong Kong, China; 13 Chennai Antiviral Research and Treatment Clinical Research Site (CART CRS), YRGCARE Medical Centre, VHS, Chennai, India; 14 National Center for HIV/AIDS, Dermatology & STDs, and University of Health Sciences, Phnom Penh, Cambodia; 15 Research Institute for Tropical Medicine, Manila, Philippines; 16 Hospital Sungai Buloh, Sungai Buloh, Malaysia; 17 National Hospital for Tropical Diseases, Hanoi, Vietnam; 18 Institute of Infectious Diseases, Pune, India; 19 University Malaya Medical Centre, Kuala Lumpur, Malaysia; 20 Beijing Ditan Hospital, Capital Medical University, Beijing, China; 21 Bach Mai Hospital, Hanoi, Vietnam; 22 Division of Infectious Diseases, Department of Internal Medicine, Yonsei University College of Medicine, Seoul, South Korea; 23 National Center for Global Health and Medicine, Tokyo, Japan; 24 Chiangrai Prachanukroh Hospital, Chiang Rai, Thailand; 25 Hospital Raja Perempuan Zainab II, Kota Bharu, Malaysia; 26 Faculty of Medicine Siriraj Hospital, Mahidol University, Bangkok, Thailand; 27 TREAT Asia, amfAR—The Foundation for AIDS Research, Bangkok, Thailand; 28 Department of Microbiology and Institute of Medical Research, College of Medicine, Kaohsiung Medical University, Kaohsiung, Taiwan; The University of Hong Kong, HONG KONG

## Abstract

**Background:**

We assessed the effects of hepatitis B (HBV) or hepatitis C (HCV) co-infection on outcomes of antiretroviral therapy (ART) in HIV-infected patients enrolled in the TREAT Asia HIV Observational Database (TAHOD), a multi-center cohort of HIV-infected patients in the Asia-Pacific region.

**Methods:**

Patients testing HBs antigen (Ag) or HCV antibody (Ab) positive within enrollment into TAHOD were considered HBV or HCV co-infected. Factors associated with HBV and/or HCV co-infection were assessed by logistic regression models. Factors associated with post-ART HIV immunological response (CD4 change after six months) and virological response (HIV RNA <400 copies/ml after 12 months) were also determined. Survival was assessed by the Kaplan-Meier method and log rank test.

**Results:**

A total of 7,455 subjects were recruited by December 2012. Of patients tested, 591/5656 (10.4%) were HBsAg positive, 794/5215 (15.2%) were HCVAb positive, and 88/4966 (1.8%) were positive for both markers. In multivariate analysis, HCV co-infection, age, route of HIV infection, baseline CD4 count, baseline HIV RNA, and HIV-1 subtype were associated with immunological recovery. Age, route of HIV infection, baseline CD4 count, baseline HIV RNA, ART regimen, prior ART and HIV-1 subtype, but not HBV or HCV co-infection, affected HIV RNA suppression. Risk factors affecting mortality included HCV co-infection, age, CDC stage, baseline CD4 count, baseline HIV RNA and prior mono/dual ART. Shortest survival was seen in subjects who were both HBV- and HCV-positive.

**Conclusion:**

In this Asian cohort of HIV-infected patients, HCV co-infection, but not HBV co-infection, was associated with lower CD4 cell recovery after ART and increased mortality.

## Introduction

There are similarities in the transmission routes of and risk factors for HIV, and the hepatitis B virus (HBV) and hepatitis C virus (HCV) infection[1,2], but each has a different biology and natural history of chronic infection. The prevalence of hepatitis B and C in HIV-infected individuals has been reported to be higher than that in the general population.[3,4] With faster liver disease progression in HIV patients with hepatitis B and C, declining risk of AIDS-related opportunistic infections and increased life expectancy seen in HIV-infected patients on antiretroviral therapy (ART), HBV and HCV have emerged as important causes of liver-related morbidity and mortality in these patients.[5–13] As a consequence, there has been increasing focus on diagnosis of HBV and HCV in the management of HIV-infected patients.

Yet, compliance with hepatitis screening in HIV patients prior to initiation of ART is usually poor in resource-limited settings [14,15], and the impact of HBV and HCV co-infection on ART outcomes needs to be still better understood. While HIV virological response to ART does not seem to be affected by HBV or HCV co-infection, the impact of viral hepatitis co-infection on immunological recovery after initiation of ART remains a topic of study.[16–20] The aims of our study were to assess the effects of HBV or HCV co-infection on short- and long-term outcomes following ART initiation in patients enrolled in the TREAT Asia HIV Observational Database (TAHOD), a multi-center observational research cohort in the Asia-Pacific region.

## Materials and Methods

As of December 2012, TAHOD included 7,455 HIV patients from 21 adult HIV treatment centers in 12 countries with 24,798 person-years of follow-up. Those who ever tested HBsAg positive or anti-HCV antibody positive within the duration of the study were regarded as having a history of HBV or HCV co-infection, respectively.

The following data were collected in TAHOD: (i) patient demographics and baseline characteristics: date of clinic visit, age, sex, ethnicity, HIV exposure category, date of first positive HIV test, HIV subtype, and date and result of HBV, HCV and syphilis serology; (ii) stage of disease: CD4 and CD8 count, HIV viral load, prior and new AIDS defining illnesses, date and cause of death; (iii) treatment history: prior and current antiretroviral treatments, reason for treatment changes (e.g. treatment failure, adverse events) and prophylactic treatments for opportunistic infections.

All participating clinical sites, the data management and analysis center (Kirby Institute, Australia), and the coordinating center (TREAT Asia, Thailand) obtained institutional review board approval for the study (see Acknowledgment, Ethics Statement). Written consent was obtained when required by the local review boards.

Factors associated with HBV and/or HCV co-infection were determined by univariate and multivariate logistic regression models. Immunological and virological response to ART was assessed using change in CD4 counts at 6 months after initiating ART. Factors associated with CD4 change after six months of ART and with viral load <400 after 12 months of ART were also determined. Patients were included in the later analysis if they had both baseline CD4 counts and CD4 counts at 180 days (90–270 days) after treatment initiation available. Baseline CD4 count was obtained before initiation of ART, with the value closest to ART start within a six month window used.

Factors associated with overall survival were determined by univariate and multiple Cox proportional hazard models. All patients who were followed up were included in the survival analysis. Follow-up was censored at the last clinic visit for patients who survived. Survival of the HIV patients was estimated by the Kaplan Meier method and comparison of survival between subgroups was performed by the log rank test. Causes of death in TAHOD were collected through a standardized process that was previously validated by the D:A:D group. Specifically, the treating physician completed a set of forms documenting causes of death and associated clinical factors and lab test results, and the clinicians’ reports were reviewed by external investigators. Conflicting determinations of immediate and underlying causes of death were then adjudicated by a review committee.

Statistical analysis was done with SAS version 9.2 (SAS Institute Inc, Cary, NC). A two tailed *p* value of less than 0.05 was considered statistically significant.

## Results

Among 7,455 subjects enrolled in TAHOD as of December 2012. Most of the study subjects were male (70.0%) and aged between 40–50 years (65%). The main ethnic groups were Thai (28%) and Chinese (23%). HIV infection occurred mainly by heterosexual contact (66%), homosexual contact (19%), and injecting drug use (IDU) (8%), and only 1% occurred by blood contact.

A total of 5,656 (75.9%) underwent HBsAg testing, 5,215 (70%) underwent HCV antibody testing, and 4,966 (66.6%) underwent both HBsAg and HCV antibody testing. HBsAg was positive in 591/5656 (10.4%) subjects, and HCV antibody was positive in 794/5215 (15.2%) subjects. (Table 1) Analysis of subjects undergoing both HBV and HCV testing showed that 3,816 subjects were HBV- and HCV-negative, 427 were HBV-positive and HCV-negative, 635 were HCV-positive and HBV-negative, and 88 were HBV- and HCV-positive.

**Table 1:** Factors associated with HBV and HCV coinfection.

	HBV						HCV					
	HBsAg positive (%)	Univariate		Multivariate			HCV positive (%)	Univariate		Multivariate		
		OR	p	OR	p	95%CI		OR	p	OR	p	95%CI
Total	591/5656 (11)						794/5215 (15)					
Sex												
Male	463/3949 (12)						681/3676 (19)					
Female	127/1702 (7)	0.61	<0.001	0.66	<0.001	(0.52, 0.82)	112/1534 (7)	0.35	<0.001	0.51	<0.001	(0.40, 0.66)
Change	1/5 (20)	1.88	0.57	2.08	0.52	(0.23, 19.02)	1/5 (20)	1.09	0.93	0.99	0.99	(0.05, 21.80)
Age (years)												
≦30	159/1439 (11)						237/1332 (18)					
31–50	371/3644 (10)	0.91	0.36	0.94	0.54	(0.77, 1.15)	524/3353 (16)	0.85	0.07	1.06	0.63	(0.84, 1.33)
>50	61/573 (11)	0.96	0.79	1.01	0.96	(0.73, 1.39)	33/530 (6)	0.31	<0.001	0.54	0.01	(0.35, 0.84)
Mode of infection												
Heterosexual contact	352/3737 (9)						271/3322 (8)					
Homosexual contact	138/1059 (13)	1.45	<0.001	1.28	0.04	(1.01, 1.64)	43/1033 (4)	0.49	<0.001	0.51	<0.001	(0.35, 0.73)
Injecting drug use	65/489 (13)	1.48	0.01	1.30	0.10	(0.96, 1.74)	405/508 (80)	44.26	<0.001	34.12	<0.001	(26.16, 44.94)
Blood products	7/56 (12)	1.38	0.44	1.25	0.59	(0.56, 2.80)	29/55 (53)	12.55	<0.001	15.18	<0.001	(8.57, 27.10)
Other	29/315 (9)	0.97	0.90	0.86	0.46	(0.57, 1.29)	46/297 (15)	2.05	<0.001	2.03	<0.001	(1.42, 2.89)
Baseline CD4 count												
≦50	194/1573 (12)						282/1487 (19)					
51–200	208/1954 (11)	0.84	0.12	0.82	0.08	(0.66, 1.02)	259/1797 (14)	0.72	<0.001	0.76	0.03	(0.60, 0.98)
201–350	96/1128 (9)	0.66	<0.001	0.59	<0.001	(0.45, 0.78)	117/997 (12)	0.57	<0.001	0.87	0.37	(0.63, 1.19)
351–500	19/156 (12)	0.99	0.96	0.80	0.41	(0.47, 1.35)	5/143 (3)	0.15	<0.001	0.31	0.02	(0.11, 0.85)
≧501	7/59 (12)	0.96	0.91	0.85	0.70	(0.37, 1.95)	8/52 (15)	0.78	0.52	1.20	0.73	(0.44, 3.22)
Not tested	67/786 (9)	0.66	0.01	0.58	<0.001	(0.43, 0.80)	123/739 (17)	0.85	0.18	0.55	<0.001	(0.40, 0.77)
Baseline HIV viral load												
40–20000	68/606 (11)						52/550 (9)					
20001–80000	79/692 (11)	1.02	0.91	0.98	0.92	(0,69, 1.40)	49/634 (8)	0.80	0.29	0.73	0.19	(0.45, 1.17)
80001–200000	65/694 (9)	0.82	0.27	0.76	0.16	(0.53, 1.11)	57/641 (9)	0.93	0.74	0.68	0.12	(0.42, 1.11)
>200000	98/910 (11)	0.95	0.78	0.89	0.48	(0.63, 1.25)	78/810 (10)	1.02	0.91	0.85	0.48	(0.55, 1.33)
Not tested	281/2754 (10)	0.90	0.46	0.94	0.70	(0.69, 1.28)	558/2580 (22)	2.64	<0.001	1.88	<0.001	(1.29, 2.76)
CDC stage												
CDC A	288/2771 (10)						345/2562 (13)					
CDC B	77/679 (11)	1.11	0.47	1.11	0.46	(0.84, 1.46)	73/601 (12)	0.89	0.39	0.90	0.5	(0.65, 1.27)
CDC C	226/2206 (10)	0.98	0.86	0.86	0.15	(0.70, 1.06)	376/2052 (18)	1.45	<0.001	0.83	0.1	(0.66, 1.04)
Prior mono/dual ARV												
No	550/5236 (11)						749/4843 (15)					
Yes	41/420 (10)	0.92	0.63	0.90	0.52	(0.63, 1.26)	45/372 (12)	0.76	0.08	0.67	0.07	(0.44, 1.03)
HIV-1 subtype												
B	19/134 (14)						6/161 (4)					
01AE	8/76 (11)	0.71	0.45	0.84	0.70	(0.34, 2.05)	2/72 (3)	0.74	0.71	0.45	0.39	(0.07, 2.83)
07BC	0/2 (0)	0	0.96	0	0.96	(0, Inf)	0/2 (0)	0	0.98	0	0.99	(0, Inf)
C	1/5 (20)	1.51	0.72	1.68	0.66	(0.17, 16.90)	0/5 (0)	0	0.97	0	0.98	(0, Inf)
Not tested	563/5439 (10)	0.70	0.15	0.79	0.35	(0.47, 1.31)	786/4975 (16)	4.85	<0.001	1.90	0.16	(0.77, 4.64)

Table 1 summarizes the factors associated with HBV or HCV co-infection. In multivariate analysis, female sex was associated with a lower risk of HBV infection (odds-ratio (OR) = 0.66, 95% confidence interval (CI) = 0.52–0.82). When compared with heterosexual risk of HIV infection, homosexual contact was associated with an increased likelihood of HBV co-infection (OR = 1.28, 95%CI = 1.01–1.64). When compared to subjects with baseline CD4 counts <50 cells/μL, those with CD4 counts in the 201–350 cells/μL range showed a decreased risk of HBV infection (OR = 0.59, 95%CI = 0.45–0.78).

Females had also a lower risk of HCV infection than males (OR = 0.51, 95%CI = 0.40–0.66). When compared to heterosexual risk of HIV infection, homosexual contact was associated with a lower risk of HCV co-infection (OR = 0.51, 95%CI = 0.36–0.73), whereas IDU (OR = 34.12, 95%CI = 26.16–44.94), history of receiving blood products (OR = 15.18, 95%CI = 8.57–27.10) and other routes of HIV infection (OR = 2.03, 95%CI = 1.42–2.89) were associated with a higher risk of HCV co-infection. When compared with subjects with baseline CD4 counts <50 cells/μL, those with counts in the 50–200 cells/μL range (OR = 0.76, 95%CI = 0.60–0.98) and in the 351–500 cells/μL range (OR = 0.31, 95%CI = 0.11–0.85) were at decreased risk of HCV co-infection.

CD4 counts were available at baseline and 6 months of ART in 4,795 subjects. (Table 2) Six-month CD4 counts increased by a mean of 122.4 cells/μL (sd = 114.8 cells/μL). After 6 months of ART, CD4 counts remained unchanged in subjects whose baseline CD4 was over 500 cells/μL, while CD4 counts increased by 80 cells/μL in subjects whose baseline CD4 counts were under 500 cells/μL. In multivariate analysis, HBV positive subjects had a smaller increase in CD4 count than HBV negative subjects, but this association was not significant (difference = -10.17, p = 0.08). HCV positive subjects had a smaller increase in CD4 count than HCV negative subjects (difference = -23.91, p<0.001). Smaller increases in CD4 counts were seen in subjects aged over 50 years compared to subjects aged 30 or younger (difference = -17.88, p = 0.004) and subjects reporting HIV infection through IDU compared to subjects reporting heterosexual contact (difference = -22.11, p = 0.005). Subjects with baseline CD4 count 51–350 cells/μL had greater increases (CD4 count = 51–200, difference = 20.08, p<0.001; CD4 count = 201–350, difference = 22.23, p<0.001) and those with baseline CD4 count >350 cells/μL had smaller increases (CD4 count = 351–500, difference = -26.36, p = 0.004; CD4 count >500, difference = -113.42, p<0.001) in CD4 counts than subjects with baseline CD4 count ≤50 cells/μL. Greater increases in CD4 counts were seen in subjects with baseline HIV viral load >20,000 copies/mL compared to subjects with viral load <20,000 copies/mL (viral load = 20,001–80,000, difference = 23.11; viral load = 80,001–200,000, difference = 35.11; viral load ≥200,000, difference = 47.94; all p<0.001). Subjects with HIV subtype 01AE (difference = -35.47, p = 0.03) and subtype C (difference = -169.64, p = 0.003) had smaller increases in CD4 counts than subjects with subtype B.

**Table 2:** Factors associated with CD4 change after six months of ART.

	No.	Mean change±sd	Univariate		Multivariate		
			difference	p	difference	p	95%CI
Total	4795	122.4±114.8					
HBsAg testing							
Negative	3365	121.8±109.8					
Positive	414	109.9±102.8	-11.90	0.05	-10.17	0.08	(-21.56, 1.22)
Not tested	1016	129.6±133.8	7.84	0.06	9.29	0.10	(-1.77, 20.35)
HCV antibody testing							
Negative	3010	124.6±109.8					
Positive	512	90.3±93.8	-34.27	<0.001	-23.91	<0.001	(-36.52, -11.30)
Not tested	1273	130.0±131.0	5.43	0.16	3.83	0.47	(-6.48, 14.13)
Sex							
Male	3329	122.3±115.3					
Female	1463	122.8±113.6	0.50	0.89	-0.42	0.91	(-7.91, 7.06)
Change	3	0±152.0	-122.32	0.07	-125.77	0.05	(-251.88, 0.34)
Age (years)							
≦30	1134	126.2±117.3					
31–50	3179	122.3±114.5	-3.88	0.33	-6.12	0.12	(-13.80, 1.55)
>50	482	114.3±111.0	-11.87	0.06	-17.88	0.004	(-30.01, -5.74)
Mode of infection							
Heterosexual contact	3306	123.6±112.6					
Homosexual contact	794	131.5±122.9	7.91	0.08	7.34	0.15	(-2.65, 17.33)
Injecting drug use	316	82.7±100.7	-38.94	<0.001	-22.11	0.005	(-37.59, -6.63)
Blood products	40	119.7±139.3	-3.87	0.83	3.70	0.84	(-31.33, 38.73)
Other	339	124.8±120.1	1.15	0.86	-0.83	0.90	(-13.60, 11.94)
Baseline CD4 count							
≦50	1484	114.0±74.6					
51–200	1921	132.7±115.8	18.68	<0.001	20.08	<0.001	(12.21, 27.94)
201–350	1113	132.8±127.4	18.80	<0.001	22.23	<0.001	(12.58, 31.88)
351–500	188	84.2±166.1	-29.82	0.001	-26.36	0.004	(-44.17, -8.55)
≧501	89	-7.8±208.2	-121.79	<0.001	-113.42	<0.001	(-138.21, -88.63)
Baseline HIV viral load							
40–20,000	669	86.7±180.2					
20,001–80,000	618	121.4±107.6	34.72	<0.001	23.11	<0.001	(10.73, 35.49)
80,001–200,000	570	131.7±109.4	44.97	<0.001	35.11	<0.001	(22.31, 47.91)
>200,000	795	148.2±132.6	61.51	<0.001	47.94	<0.001	(36.04, 59.84)
Not tested	2145	120.7±110.6	34.00	<0.001	28.04	<0.001	(17.57, 38.50)
CDC stage							
CDC A	2433	121.6±120.0					
CDC B	524	116.2±104.3	-5.43	0.33	-7.28	0.19	(-18.06, 3.50)
CDC C	1838	125.2±110.6	3.56	0.32	4.25	0.27	(-3.31, 11.81)
HIV-1 subtype							
B	144	144.1±132.4					
01AE	78	111.6±76.7	-32.4	0.04	-35.47	0.03	(-66.69, -4.25)
07BC	2	188.5±46.0	44.45	0.59	33.44	0.67	(-121.84, 188.72)
C	4	-30.0±233.9	-174.05	0.003	-169.64	0.003	(-280.27, -59.02)
Not tested	4567	122.0±114.6	-22.05	0.02	-20.52	0.04	(-39.72, -1.33)

In multivariate analysis, factors found to be associated with viral load <400 after 12 months of ART included age 31–50 (OR = 1.68, 95%CI = 1.24–2.28), age >50 (OR = 2.27, 95%CI = 1.37–3.77), mode of infection other than heterosexual or homosexual contact (OR = 0.66, 95%CI = 0.44–0.97), baseline CD4 count 201–350 (OR = 1.92, 95%CI = 1.27–2.90), baseline HIV viral load 80,001–200,000 (OR = 0.60, 95%CI = 0.39–0.90), ART antiretroviral regimen containing nucleoside reverse transcriptase inhibitor and protease inhibitor (OR = 0.55, OR = 0.41–0.73), prior ART (OR = 0.28, 95%CI = 0.19–0.39), and HIV-1 subtype C (OR = 0.12, 95%CI = 0.02–0.94) (Table 3). There was no association between HBV or HCV co-infection with virological response.

**Table 3:** Factors associated with viral load suppression <400 at 12 months after initiating ART.

	No. of cases	No. of cases	No. of	Univariate		Multivariate		
	with VL suppression	without VL suppression	cases	OR	p	OR	p	95%CI
Total	2410(89.29)	289(10.71)	2699					
HBsAg testing								
Negative	1739 (72.16)	210 (72.66)	1949(72.21)					
Positive	217 (9.00)	21 (7.27)	238(8.82)	1.33	0.25	1.61	0.07	(0.96,2.69)
Not tested	454 (18.84)	58 (20.07)	512(18.97)	0.96	0.82	1.08	0.73	(0.70,1.68)
HCV antibody testing								
Negative	1620 (67.22)	196 (67.82)	1816(67.28)					
Positive	132 (5.48)	19 (6.57)	151(5.59)	0.82	0.43	0.85	0.55	(0.49,1.47)
Not tested	658 (27.30)	74 (25.61)	732(27.12)	1.13	0.39	1.1	0.65	(0.74,1.64)
Sex								
Male	1768 (73.36)	226 (78.20)	1994(73.88)					
Female	640 (26.56)	62 (21.45)	702(26.01)	1.31	0.08	1.13	0.48	(0.81,1.58)
Change	2 (0.08)	1 (0.35)	3(0.11)	0.25	0.26	0.34	0.4	(0.03,4.21)
Age (years)								
≦30	485 (20.12)	79 (27.34)	564(20.90)					
31–50	1635 (67.84)	186 (64.36)	1821(67.47)	1.42	0.02	1.68	0.0009	(1.24,2.28)
>50	290 (12.03)	24 (8.30)	314(11.63)	1.88	0.01	2.27	0.0015	(1.37,3.77)
Mode of infection								
Heterosexual contact	1470 (61.00)	166 (57.44)	1636(60.62)					
Homosexual contact	661 (27.43)	77 (26.64)	738(27.34)	0.98	0.89	0.95	0.79	(0.67,1.35)
Other	279 (11.58)	46 (15.92)	325(12.04)	0.68	0.03	0.66	0.04	(0.44,0.97)
CDC stage								
CDC A	1323 (54.90)	134 (46.37)	1457(53.98)					
CDC B	297 (12.32)	24 (8.30)	321(11.89)	1.18	0.49	1.26	0.36	(0.77,2.05)
CDC C	790 (32.78)	131 (45.33)	921(34.12)	0.6	0.0001	0.75	0.07	(0.55,1.02)
Baseline CD4 count								
≦50	652 (27.05)	110 (38.06)	762(28.23)					
51–200	908 (37.68)	106 (36.68)	1014(37.57)	1.44	0.01	1.26	0.15	(0.92,1.73)
201–350	666 (27.63)	48 (16.61)	714(26.45)	2.32	< .0001	1.92	0.0021	(1.27,2.90)
351–500	128 (5.31)	15 (5.19)	143(5.30)	1.56	0.15	1.65	0.15	(0.84,3.22)
≧501	56 (2.32)	10 (3.46)	66(2.45)	0.89	0.74	0.97	0.94	(0.43,2.17)
Baseline HIV viral load								
40–20000	581 (24.11)	61 (21.11)	642(23.79)					
20001–80000	579 (24.02)	63 (21.80)	642(23.79)	1	0.99	0.81	0.31	(0.54,1.22)
80001–200000	526 (21.83)	72 (24.91)	598(22.16)	0.79	0.21	0.6	0.01	(0.39,0.90)
>200000	724 (30.04)	93 (32.18)	817(30.27)	0.86	0.38	0.69	0.07	(0.46,1.03)
ARV type								
ARV1	1779 (75.61)	167 (59.43)	1946(73.88)					
ARV2	574 (24.39)	114 (40.57)	688(26.12)	0.47	< .0001	0.55	< .0001	(0.41,0.73)
Prior mono/dual ARV								
No	2181 (90.50)	209 (72.32)	2390(88.55)					
Yes	229 (9.50)	80 (27.68)	309(11.45)	0.26	< .0001	0.28	< .0001	(0.19,0.39)
HIV-1 subtype								
B	116 (4.81)	21 (7.27)	137(5.08)					
01AE	44 (1.83)	10 (3.46)	54(2.00)	0.77	0.54	0.9	0.82	(0.36,2.28)
07BC	2 (0.08)	0 (0.00)	2(0.07)	>999.999	0.98	>999.999	0.98	(0.001,>999.999)
C	2 (0.08)	2 (0.69)	4(0.15)	0.18	0.1	0.12	0.04	(0.02,0.94)
Not tested	2246 (93.20)	256 (88.58)	2502(92.70)	1.62	0.06	1.16	0.59	(0.67,2.00)

ARV1: antiretroviral regimen containing nucleoside reverse transcriptase inhibitor + non-nucleoside reverse transcriptase inhibitor, ARV2: antiretroviral regimen containing nucleoside reverse transcriptase inhibitor + protease inhibitor

A total of 5656 patients were on ART, of whom 5130 (90.7%) were on an ART regimen containing lamivudine. Of the patients on ART, 591 (10.4%) were HBV co-infected. In these co-infected patients, ART containing lamivudine (518/591 = 87.6%) was most commonly prescribed, followed by ART containing emtricitabine (42/591 = 7.1%). Comparison of HBV co-infected patients with ART containing lamivudine with those with ART not containing lamivudine showed no statistically significant differences in CD4 change and VL suppression <400 at 12 months (data not shown). Outcomes in co-infected patients receiving the combination of lamivudine and tenofovir and those receiving emtricitabine and tenofovir were also similar (data not shown).

Factors affecting survival were evaluated in all 7,455 study subjects, contributing 24,978 person-years of prospective follow-up (Table 4). Subjects were followed for a mean of 3.33 years. During the follow-up period, 303 subjects died, and the overall mortality was 1.22 per 100 person-years. Analysis of causes of death showed that 18 subjects had AIDS-related deaths, 141 had non-AIDS-related deaths, 6 had liver-related deaths, 5 had cardiovascular disease-related deaths, and 133 died of other causes. Multivariate analysis showed that the mortality rate was higher in HCV-positive subjects (HR = 1.81, p = 0.004, 95%CI = 1.21–2.72) and lower in female subjects (HR = 0.76, p = 0.07, 95%CI = 0.57–1.02). Subjects aged over 50 years had a higher mortality rate than those under 30 years (HR = 3.87, p<0.001, 95%CI = 2.69–5.57). Subjects previously receiving ART had increased mortality when compared to treatment-naive subjects (HR = 1.48, p = 0.03, 95%CI = 1.05–2.08). The higher the baseline CD4 count, the lower the mortality rate (MR = 2.01/100 person-years for CD4 count ≤50, MR = 0.68/100 person-years for CD4 count >500); and the higher the baseline HIV viral load, the higher the mortality rate (MR = 0.65 for viral load ≤20,000, MR = 1.62 for viral load >200,000).

**Table 4:** Factors associated with mortality after entry to TAHOD.

	No. of	No. of	Rate	Univariate		Multivariate		
	patients	deaths	(/100 py)	HR	p	HR	p	95%CI
Total	7455	303	1.22					
HBsAg testing								
Negative	5065	170	1.02					
Positive	591	29	1.48	1.45	0.06	1.33	0.15	(0.90, 1.98)
Not tested	1799	104	1.68	2.17	<0.001	2.17	<0.001	(1.52, 3.10)
HCV antibody testing								
Negative	4421	154	1.01					
Positive	794	46	2.53	2.22	<0.001	1.81	0.004	(1.21, 2.72)
Not tested	2240	103	1.33	1.34	0.02	0.86	0.43	(0.60, 1.25)
Sex								
Male	5245	241	1.36					
Female	2202	60	0.85	0.61	<0.001	0.76	0.07	(0.57, 1.02)
Change	6	2	10.57	7.31	0.01	7.34	0.006	(1.78, 30.38)
Age (years)								
≦30	1879	53	0.92					
31–50	4855	171	1.03	1.15	0.38	1.07	0.66	(0.79, 1.47)
>50	721	79	3.28	3.64	<0.001	3.87	<0.001	(2.69, 5.57)
Mode of infection								
Heterosexual contact	4920	203	1.21					
Homosexual contact	1384	34	0.68	0.57	<0.001	0.69	0.06	(0.46, 1.02)
Injecting drug use	602	36	2.85	2.04	<0.001	1.34	0.20	(0.86, 2.08)
Other	549	30	1.75	1.41	0.08	1.39	0.11	(0.93, 2.07)
CDC stage								
CDC A	3751	87	0.73					
CDC B	835	20	0.70	0.98	0.92	0.94	0.82	(0.58, 1.54)
CDC C	2869	196	1.97	2.77	<0.001	2.10	<0.001	(1.59, 2.77)
Baseline CD4 count								
≦50	1955	125	2.01					
51–200	2558	92	1.13	0.56	<0.001	0.67	0.005	(0.51, 0.89)
201–350	1489	30	0.68	0.33	<0.001	0.51	0.002	(0.34, 0.79)
351–500	236	9	1.10	0.56	0.10	0.87	0.69	(0.43, 1.75)
≧501	118	3	0.68	0.36	0.08	0.72 72	0.58	(0.22, 2.33)
Not tested	1099	44	0.93	0.50	<0.001	0.62	0.01	(0.43, 0.91)
Baseline HIV viral load								
40–20000	866	20	0.65					
20001–80000	860	24	0.86	1.29	0.39	1.29	0.40	(0.71, 2.37)
80001–200000	833	37	1.45	2.17	0.01	1.94	0.02	(1.10, 3.42)
>200000	1127	61	1.62	2.45	<0.001	1.97	0.01	(1.16, 3.35)
Not tested	3769	161	1.28	1.98	<0.001	1.67	0.05	(1.01, 2.77)
ARV type								
ARV1	6172	246	1.33					
ARV2	1152	48	0.85	0.72	0.04	0.78	0.15	(0.55, 1.10)
ARV3	131	9	1.36	1.17	0.65	1.14	0.72	(0.57, 2.29)
Prior mono/dual ARV								
No	6849	254	1.19					
Yes	606	49	1.41	1.37	0.05	1.48	0.03	(1.05, 2.08)
HIV-1 subtype								
B	192	6	0.54					
01AE	86	2	0.41	0.75	0.72	0.58	0.52	(0.12, 2.95)
07BC	2	0	0	0	1.00	0	1.00	(0, Inf)
C	5	0	0	0	0.99	0	0.99	(0, Inf)
Not tested	7170	295	1.27	2.08	0.08	1.71	0.99	(0.74, 3.94)

ARV1: antiretroviral regimen containing nucleoside reverse transcriptase inhibitor + non-nucleoside reverse transcriptase inhibitor, ARV2: antiretroviral regimen containing nucleoside reverse transcriptase inhibitor + protease inhibitor, ARV3: other antiretroviral regimens

**Fig 1:**
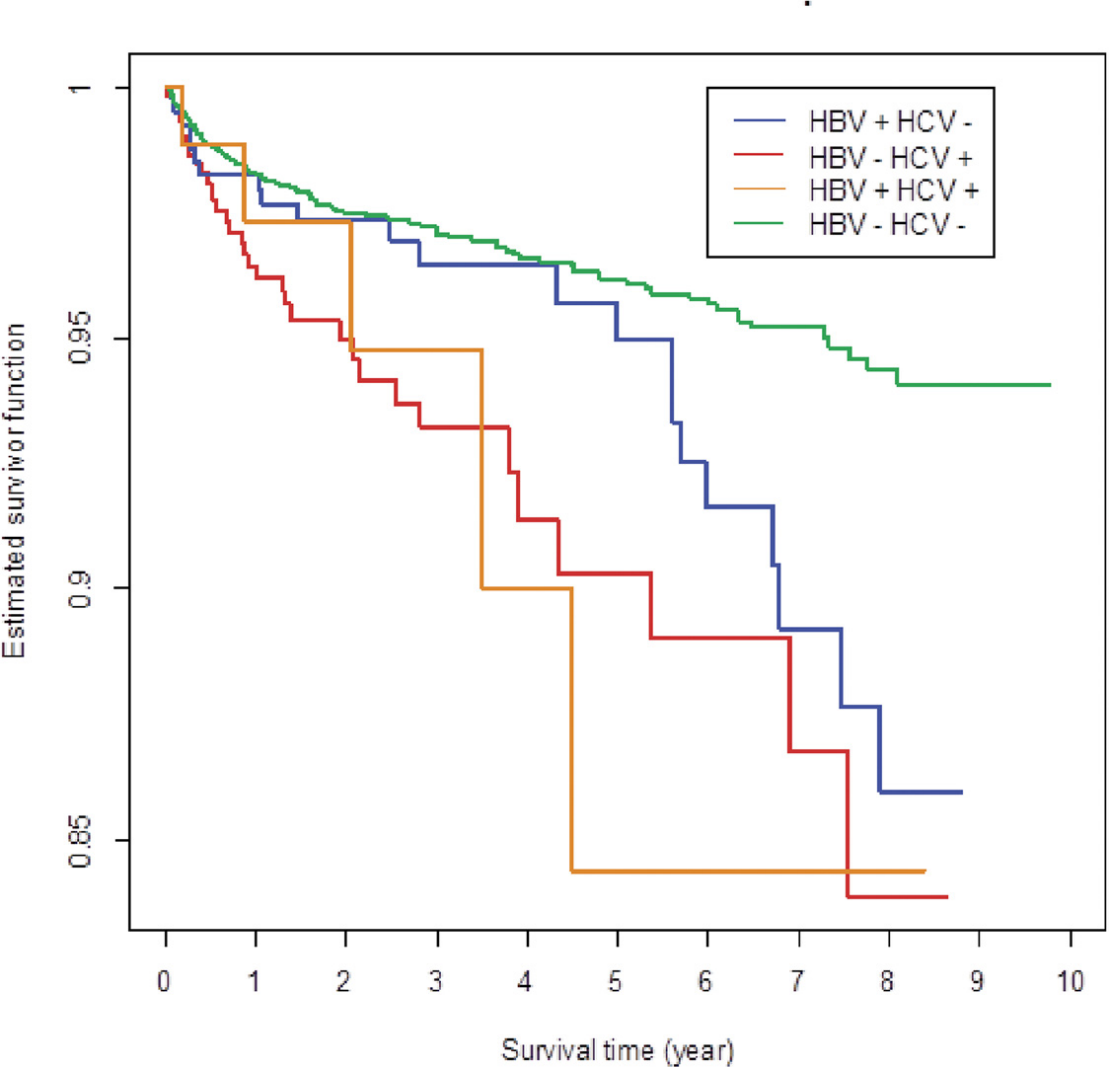
Overall survival by HBV or HCV co-infection status.

Survival was shortest in subjects who were both HBV- and HCV-positive, and survival was worse in HCV-positive than in HBV-positive subjects (Fig 1).

## Discussion

This study determined the effects of HBV or HCV co-infection on short- and long-term outcomes following ART in patients enrolled in TAHOD. Results showed that risk factors affecting CD4 change 6 months after ART included HCV co-infection, older age, injecting drug use, lower baseline CD4 count, higher HIV viral load, and HIV-1 subtypes 01AE and C. Risk factors affecting viral load suppression to <400 12 months after ART included age, mode of transmission, baseline CD4 count, baseline viral load, ART regimen, prior ART, and HIV-1 subtype C. HBV and HCV co-infection was not associated with virological response. Significant risk factors affecting mortality included HCV co-infection, older age, late CDC stage, lower baseline CD4 count, higher baseline HIV viral load, and prior mono or dual ART.

The major limitation of this study was that we did not have sufficient data on HCV RNA to confirm the presence of chronic HCV infection. Antibody testing indicates that the patient had a history of HCV infection, which could be current or resolved. However, HCV RNA testing has rarely been available in routine HIV clinical care settings in the low- and middle-income country settings where the large majority of our patients receive care. The small number of patients who did have HCV RNA results were likely from high-income settings and/or were high-risk or had other indicators of liver disease, which makes it difficult to interpret their results in comparison to the overall cohort. Information on treatment of viral hepatitis that patients may have received beyond antiretrovirals active against HBV also was not available. In addition, as the 21 HIV treatment centers participating in TAHOD are mainly large urban referral hospitals, results from this study cannot be generalized to patients from different settings, notably rural areas, who may have a more limited access to medical care and disease monitoring.

Another limitation of this study was that the timing of the hepatitis screening in relationship to outcome or ART initiation were at different times. The HBV and HCV test results reflected ever being tested. There could still be some misclassification (e.g., some negatives could have become positive), but we believe this misclassification would have underestimated effects.

In this study, hepatitis B and C were detected in 10.5% and 15.2% of subjects undergoing screening, respectively. In contrast, a large African cohort of HIV-infected patients showed higher hepatitis B (17.8%) than hepatitis C (11.3%) co-infection prevalence.[21] The HIV-HBV co-infection rate reported in our study was higher than that of another multi-country study led by Thio et al., which showed HIV-HBV co-infection prevalences of 5.9% in Asia, 6.7% in Africa, 5.1% in Central/South America, and 4.8% in North America.[22] However, the latter study included only 2 Asian countries—India and Thailand. A study by Amin et al. showed that HIV-HCV co-infection prevalence in different countries varied greatly, ranging from 1.9% in South Africa to 48.6% in Italy.[23]

A previous report from TAHOD with data from 1372 subjects who had undergone hepatitis screening up to December 2005 showed that hepatitis co-infection had no effect on survival. [24] However, results from our expanded database up to December 2012 showed that shorter survival was seen in patients with co-infection. Survival was shortest in HBV- and HCV-positive subjects, and HCV-positive subjects had worse survival than HBV-positive subjects. These findings are in agreement with those of other studies that have also reported hepatitis co-infection to be associated with increased mortality in HIV patients.[25,26]

That the survival analysis identified HCV but not IDU as a risk factor of increased mortality is probably due to strong correlation between the 2 variables [27]. In our study, 80% of those with IDU as part of their HIV exposure history and serologic testing had positive HCV antibody tests. Survival and smaller increases in CD4 count among those with positive HCV antibody tests may be due to risks associated with HCV infection itself, such as IDU or other unmeasured confounders. Although adjustments for IDU were made during multivariate analysis, it was difficult to compensate for the collinearity anticipated.

CD4 counts at baseline or 6 months after ART were missing in 2660 (35.7%) of the patients. Comparison of these patients with those with both baseline and 6-month CD4 counts showed that a lower percentage of these patients who had less CD4 monitoring had previously received antiretroviral monotherapy (5.8% vs. 9.5%, p<0.001) and had been infected with HIV via heterosexual contact (60.7% vs. 69.0%, p<0.001). This potential bias is a limitation of our study. These patients were excluded from the CD4 assessments, but they were included in the overall HBV and HCV analysis.

Previous studies showed an association between lower baseline CD4 cell count and HBV co-infection [21,22,28] but no association with HCV co-infection.[21,23] Our study showed that subjects with the lowest CD4 counts had higher odds of being HBV- and HCV-positive. In addition, the increases in CD4 cell counts 6 months after ART were smaller in HBV co-infected and HCV co-infected subjects than in those with HIV only (borderline significant). The Swiss HIV Cohort Study also reported impaired CD4 cell recovery during ART in HBV co-infected individuals[29], while other large studies such as those from the EuroSIDA group[26] and from South Africa [30] did not show an association between HBV co-infection and immunological recovery during ART. A few mechanisms have been proposed to explain the impaired immunological recovery that has been seen in some studies in HBV co-infected patients: these include T-lymphocyte exhaustion [31,32], upregulation of apoptotic pathways and specifically increased T-cell apoptosis[33] that has been described with HBV active infection and replication, and also splenic sequestration of lymphocytes which may be due to HBV-related hepatic fibrosis.[29] A study by You et al. showed that treatment of HBV co-infection led to increases in CD4 cell counts[34], and the Swiss HIV Cohort Study recently showed that CD4 cell increase in patients with resolved HBV infection was similar to that in HBV-uninfected individuals.[29] Studies on the influence of HCV co-infection on HIV progression have shown inconclusive findings. However, in line with our findings, the Swiss HIV Cohort Study reported delayed CD4 cell recovery after initiation of ART in HCV co-infected patients.[25] Seminari et al. reported that HCV co-infection impaired early immunological recovery but not late immunological recovery after ART.[35]

### Ethics Statement

Ethics approval was granted for the TAHOD study design, methods and consent procedures by the University of New South Wales Human Research Ethics Committee. Site specific study governance was granted by site-relevant institutional review boards: Ministry of Health National Ethics Committee for Health Research (Cambodia), Ethical Committee of Beijing Ditan Hospital Affiliated to Capital Medical University (China), Research Ethics Committee Kowloon Central / Kowloon East, Hospital Authority IRB (China), Institutional Review Board Of YRG CARE (India), Institutional Ethics Committee Rao Nursing Home (India), Kerti Praja Foundation IRB (Indonesia), Committee of Medical Research Ethics, Faculty of Medicine University of Indonesia (Indonesia), National Center for Global Health and Medicine Human Research Ethics Committee (Japan), Medical Research & Ethics Committee, Ministry of Health (for Sungai Buloh Hospital and Hospital Raja Perempuan Zainab II, Malaysia), Medical Ethics Committee, University Malaya Medical Centre (Malaysia), Research Institute for Tropical Medicine, Department of Health (Philippines), National Healthcare Group IRB, Domain Specific Review Board (Singapore), Severance Hospital Yonsei University College of Medicine Institutional Review Board (South Korea), Institutional Review Board of Taipei Veterans General Hospital (Taiwan), The Internal Ethical Committee for Research in Human Subject, Chiangrai Prachanukroh Hospital (Thailand), Institutional Review Board Faculty of Medicine, Chulalongkorn University (Thailand), Committee on Human Rights Related to Research Involving Human Subjects Faculty of Medicine Ramathibodi Hospital, Mahidol University (Thailand), Research Ethics of the Faculty of Medicine, Chiang Mai University (Thailand), Siriraj Institutional Review Board, Mahidol University (Thailand), Ministry of Health, Hanoi School of Public Health IRB (Vietnam), and National Hospital of Tropical Diseases IRB (Vietnam).

Written informed consent was not sought in TAHOD unless required by a site’s local institutional review board. The need for written consent was waived by the following ethics committees: Ministry of Health National Ethics Committee for Health Research (Cambodia), Ethical Committee of Beijing Ditan Hospital Affiliated to Capital Medical University (China), Research Ethics Committee Kowloon Central / Kowloon East, Hospital Authority IRB (China), Institutional Review Board Of YRG CARE (India), Institutional Ethics Committee Rao Nursing Home (India), Kerti Praja Foundation IRB (Indonesia), Committee of Medical Research Ethics, Faculty of Medicine University of Indonesia (Indonesia), Medical Research & Ethics Committee, Ministry of Health (for Sungai Buloh Hospital, Malaysia), Medical Ethics Committee, University Malaya Medical Centre (Malaysia), The Internal Ethical Committee for Research in Human Subject, Chiangrai Prachanukroh Hospital (Thailand), Ministry of Health, Hanoi School of Public Health IRB (Vietnam), and National Hospital of Tropical Diseases IRB (Vietnam).

## Conclusion

In this Asian regional HIV cohort, HBV or HCV co-infection by serological testing was common. Patients with HCV co-infection had significantly lower CD4 counts, worse CD4 cell recovery after 6 months of ART, and poorer survival than HIV mono-infected patients. These findings confirm the importance of screening for HBV and HCV co-infection and increasing access to confirmatory hepatitis testing, and support recommendations for use of antiretrovirals active against HBV in ART regimens for co-infected patients.
